# Transorbital approach for endoscopic repair of encephalocele

**DOI:** 10.3171/2020.4.FocusVid.19907

**Published:** 2020-04-01

**Authors:** Christina E. Sarris, Griffin D. Santarelli, Andrew S. Little

**Affiliations:** Department of Neurosurgery, Barrow Neurological Institute, St. Joseph’s Hospital and Medical Center, Phoenix, Arizona

**Keywords:** cerebrospinal fluid, encephalocele, endoscopy, rhinorrhea, transorbital, video

## Abstract

This video demonstrates the transorbital approach for endoscopic repair of an anterior skull base encephalocele. The patient is a 77-year-old man with morbid obesity and a 2-year history of left-sided cerebrospinal fluid (CSF) rhinorrhea and radiographic evidence of an anterior skull base defect with an encephalocele. An endoscopic transorbital approach was chosen for repair because of its minimally invasive access to the anterolateral skull base. The patient had an excellent clinical outcome with resolution of the CSF rhinorrhea and preservation of full vision and extraocular muscle function.

The video can be found here: https://youtu.be/oDhZgnaiZ00.

## Transcript

We will be demonstrating a transorbital approach for endoscopic repair of an anterior skull base encephalocele.

The patient is a 77-year-old man with a 2-year history of left-sided nasal drainage. The drainage had a salty, metallic taste and was confirmed beta-2 transferrin positive. He had no prior craniofacial trauma or sinus surgery. His past medical history was significant for obesity, obstructive sleep apnea, and hemidiaphragm paralysis. He had a normal physical exam, including nasal endoscopy.

CT of the head demonstrated thinning and erosion of the left orbital roof, or floor of the left anterior cranial fossa. An MRI of the brain demonstrated frontal lobe herniation into the defect seen on CT. Findings were consistent with an anterior skull base encephalocele. His risk factors for development of the encephalocele included morbid obesity and sleep apnea, and the etiology for its formation was suspected to be intracranial hypertension.

Surgical repair was recommended. Preoperative intracranial pressure monitoring was not considered in the setting of an active cerebrospinal fluid leak. As well, based on the author’s experience, lumbar drainage or other spinal fluid diversion was not performed prophylactically or as an adjuvant for surgical repair. Both open and endoscopic techniques were discussed with the patient; an endoscopic approach was favored for its less invasive access to the skull base compared to a craniotomy. The transorbital approach was chosen for its access to the anterolateral skull base. The surgical plan was for repair via the left endoscopic transorbital approach with endoscopic endonasal harvest of a free mucosal graft from the middle turbinate.

The surgical team included an attending neurosurgeon and otolaryngologist, both with subspecialty training in skull base surgery. The otolaryngologist performed the approach to the anterior skull base, and the neurosurgeon isolated and reduced the encephalocele. Both surgeons were involved in the multilayered reconstructive closure.

The patient was positioned supine on the operating room table and intubated under general endotracheal anesthesia **(2:35)**. The image guidance system was registered and verified. The patient was then prepped and draped in the usual sterile fashion. A tarsorrhaphy stitch was placed to keep the globe protected during the procedure. Then, an upper blepharoplasty incision was then made along the left eyelid. Dissection was carried down through the subcutaneous tissue and orbicularis muscle down to the level of the tarsal plate.

The dissection then continued toward the level of the superior orbital rim and anterior cranial fossa. The endoscope was introduced, and the periorbita was protected using a malleable retractor. The superior orbital rim was removed with a high-speed drill **(3:20)**. This step provided access to the anterior frontal sinus. With the aid of image guidance, the drilling was extended laterally until the laterally based posterior table encephalocele was encountered. The defect was approximately 1 cm in size. The bone and soft tissues surrounding the encephalocele were circumferentially dissected. The neck of the encephalocele was isolated **(4:23)**, then the herniating arachnoid was reduced **(4:37)**. Hemostasis was achieved. The free endonasal mucosal graft was harvested at this time. This was harvested from the nasal middle turbinate. Inlay dural substitute graft was then placed in the intracranial defect, followed by a rigid buttress in the epidural space to bolster the reconstruction **(5:33)**. The mucosal graft was then overlaid as the final layer for reconstruction **(5:56)**. The site was then packed with a small amount of nasal packing and dural substitute. There was no CSF leak observed at this point. The incision was then closed in a multilayer fashion **(6:07)**.

The patient remained neurologically intact postoperatively with full function of extraocular muscles and preserved vision. He had a prolonged hospital stay secondary to postoperative respiratory issues. At 4 weeks of follow-up, he was progressing well and healing appropriately, with preserved vision and extraocular muscle function. His CSF rhinorrhea had resolved.^1–5^

## Acknowledgments

The authors thank the staff of Neuroscience Publications at Barrow Neurological Institute for assistance with manuscript and video preparation.
